# Cross-Linguistic Trade-Offs and Causal Relationships Between Cues to Grammatical Subject and Object, and the Problem of Efficiency-Related Explanations

**DOI:** 10.3389/fpsyg.2021.648200

**Published:** 2021-07-12

**Authors:** Natalia Levshina

**Affiliations:** Neurobiology of Language Department, Max Planck Institute for Psycholinguistics, Nijmegen, Netherlands

**Keywords:** efficiency, trade-offs, causal networks, subject, object

## Abstract

Cross-linguistic studies focus on inverse correlations (trade-offs) between linguistic variables that reflect different cues to linguistic meanings. For example, if a language has no case marking, it is likely to rely on word order as a cue for identification of grammatical roles. Such inverse correlations are interpreted as manifestations of language users’ tendency to use language efficiently. The present study argues that this interpretation is problematic. Linguistic variables, such as the presence of case, or flexibility of word order, are aggregate properties, which do not represent the use of linguistic cues in context directly. Still, such variables can be useful for circumscribing the potential role of communicative efficiency in language evolution, if we move from cross-linguistic trade-offs to multivariate causal networks. This idea is illustrated by a case study of linguistic variables related to four types of Subject and Object cues: case marking, rigid word order of Subject and Object, tight semantics and verb-medial order. The variables are obtained from online language corpora in thirty languages, annotated with the Universal Dependencies. The causal model suggests that the relationships between the variables can be explained predominantly by sociolinguistic factors, leaving little space for a potential impact of efficient linguistic behavior.

## Some Problems With Efficient Trade-Offs

In recent years there have been quite a few cross-linguistic studies that investigate trade-offs between different communicative or cognitive costs. It is often claimed that these trade-offs are explained by the need to support efficient communication. For example, Kemp et al. (2018) argue that lexical systems of kinship words or color terms demonstrate a trade-off between cognitive costs (number of rules needed to describe a system) and communicative costs (divergence between the probability distributions of the speaker and the addressee). Coupé et al. (2019) find a trade-off between information rate and speech rate, which, on the one hand, saves language users from cognitive overload, and helps to save time, on the other hand.

Similarly, Koplenig et al. (2017) demonstrate a trade-off between information conveyed by word order and word structure, represented by information-theoretic measures and based on corpus data from almost 1,000 languages. Isolating languages, such as Mandarin Chinese, have high scores on information conveyed by word order, but low scores on information carried by word structure. In contrast, polysynthetic languages, such as Ojibwa and Greenlandic Inuktitut, have high word structure scores, but low word order scores. Koplenig et al. (2017) interpret this correlation as an efficient trade-off: Language users can dispense with morphological marking when word order provides sufficient information about the message.

A more specific trade-off is related to the expression of grammatical subject. Berdicevskis et al. (2020) provide typological data showing that languages that have subject indexing (verbal affixes and clitics) more frequently allow for omission of subject pronouns, although this trend is not supported in Eurasia. They also use corpora of East Slavic languages to show that that the absence of person indexation in past tense encourages speakers to encode accessible subject referents by independent pronouns significantly more often (note that this tendency is also observed in some other Slavic languages, where person is always marked). The results are interpreted in terms of efficiency: Information should be conveyed linguistically, but redundancy is undesirable.

Inverse correlations between different linguistic variables have enjoyed considerable attention in research on linguistic complexity. For example, Fenk-Oczlon and Fenk (2008) argue for the following trade-offs between different language subsystems:

• Phonological complexity (e.g., large phonemic inventory, complex syllable structure, and high number of syllable types) vs. morphological complexity (e.g., high number of morphemes per word and low number of monosyllabic words);
• Morphological complexity (see above) vs. semantic complexity (polysemy and synonymy);
• Semantic complexity (see above) vs. word order complexity (e.g., flexible word order, which has low predictability and implies that language users have to learn many additional stylistic rules).^1^

As an illustration, compare English and Russian. English has a higher number of syllable types, shorter words with fewer morphemes, higher lexical and grammatical ambiguity and rigid word order. In contrast, Russian has fewer syllable types, longer words with more morphemes, lower ambiguity and more flexible word order. At least some of these trade-offs can be interpreted in terms of efficiency. The trade-off between phonological and morphological complexity is in accordance with Menzerath–Altmann’s law (Altmann, 1980), which predicts an inverse correlation between word length and syllable length. Stave et al. (2021) argue that this trade-off is efficient: it allows language users to save costs needed for working memory and planning. The trade-off between semantic and word-order complexity can be explained by the fact that ambiguous words rely on their context for assignment of lexico-semantic and grammatical properties (cf. Piantadosi et al., 2012; Hawkins, 2019).

An assumption behind these and similar claims is that language users tend to avoid both linguistic overspecification and underspecification when expressing certain information. This tendency can be interpreted as rational and efficient behavior. So, one might expect that different types of linguistic cues that express similar information will be negatively correlated. And the other way round, negative correlations could be interpreted as a sign of efficient behavior.

These assumptions are not as self-evident as they may seem, however. First of all, aggregate variables, such as the presence of case marking or flexible word order in a language, do not take into account the joint distribution of cues in usage contexts. While this lack of information may be irrelevant for languages with categorical values on linguistic variables (e.g., total lack of case marking vs. obligatory case marking without case syncretism; or perfectly rigid vs. completely random word order), this creates problems for languages with in-between values, such as optional or differential case marking, or a dominant but not exclusive word order. In fact, these are the majority of languages (e.g., Sinnemäki, 2014a; Levshina, 2019). In this case, there is a possibility of one clause containing two or zero cues, which means overspecification or underspecification, respectively. A trade-off at the aggregate level can mask these uses. Therefore, not all inverse correlations between linguistic variables representing different cues can be interpreted as a sign of efficient behavior.

Second, an inverse correlation between two linguistic variables can disappear or become weaker if we control for a third variable (e.g., Levshina, 2020a). Most importantly, we need to control for the role of accessibility of information from context in a broad sense (that is, including linguistic context, situational, and encyclopedic information), which itself is in a trade-off relationship with the amount of linguistic coding required. This trade-off has been observed in studies of phonological reduction (Jurafsky et al., 2001; Aylett and Turk, 2004; Cohen Priva, 2008; Seyfarth, 2014; Jaeger and Buz, 2017; Hall et al., 2018). In the lexicon, there is a correlation between predictability (defined in different ways) and word length (Zipf, 1965[1935]; Manin, 2006; Piantadosi et al., 2011; Mahowald et al., 2013). The length of referential expressions is known to depend on their accessibility (Ariel, 1990), which is determined by common ground (Clark and Wilkes-Gibbs, 1986). As for morphosyntactic coding asymmetries and splits, it is well known that more predictable grammatical meanings are expressed by shorter forms (including zero) than less predictable ones (e.g., Jäger, 2007; Kurumada and Jaeger, 2015; Kurumada and Grimm, 2019; Haspelmath, 2021). Lemke et al. (2021) demonstrate that fragments (i.e., incomplete sentential structures) encoding events known from everyday scripts and scenarios are perceived as more natural than fragments encoding unpredictable events. See more examples in Hawkins (2004), Jaeger and Tily (2011), and Gibson et al. (2019). That is, if some meaning is highly predictable from context or in general, it is efficient to use no overt cues at all^2^. For example, it is known that the subject of canonical imperatives does not have to be overtly expressed in the vast majority of the world’s languages, especially if the addressee is singular (Aikhenvald, 2010). If some meaning is difficult to retrieve, it may be perfectly efficient to use multiple cues. For instance, the use of resumptive pronouns, as in Hebrew and Cantonese, in certain types of relative clauses can be efficient because it makes processing easier in structurally more complex environments (Hawkins, 2004). Another case is clitic doubling in some high-contact varieties, such as languages of the Balkan Sprachbund, which means that some objects are expressed twice^3^. According to Wiemer and Hansen (2012: 127), it helps “speakers in multilingual settings of a primarily oral culture … to achieve the most reliable degree of mutual intelligibility.” So, a negative correlation between *linguistic* cues does not tell us much about efficiency if other factors, such as predictability and ease of processing, are not controlled for.

Moreover, the use of linguistic cues is multifunctional. For example, in addition to helping to identify main grammatical roles, constituent order can also allow language users to manage information structure, to facilitate production by putting accessible elements first (e.g., Bock and Warren, 1985; Ferreira and Yoshita, 2003), to maximize early access to semantic and grammatical structure (Hawkins, 2004), to save memory costs by minimizing dependency distances or syntactic domains (Hawkins, 2004; Ferrer-i-Cancho, 2006; Liu, 2008; Futrell et al., 2015), and so on. There is also a claim (Maurits, 2011) that constituent orders that frequently occur in the world’s languages make information density more uniform, avoiding peaks and troughs (Jaeger, 2006; Levy and Jaeger, 2007). This means that the overall communicative efficiency of a certain language system depends on multiple parameters, which need to be taken into account.

In addition, language users’ communicative preferences are not the only factor that shapes language structure. An important role is played by analogy (Haspelmath, 2014) and by diverse frequency effects (Bybee, 2010). In addition, many language changes are attributed to sociolinguistic factors. Under normal circumstances, for example, languages tend to accumulate morphological complexity (Dahl, 2004), but an increase in the proportion of adult L2 speakers and population size can lead to simplification and loss of inflectional morphology (McWhorter, 2011). Cross-linguistic studies reveal inverse relationships between morphological complexity and population size (Lupyan and Dale, 2010) and proportion of L2 speakers (Bentz and Winter, 2013). Fenk-Oczlon and Pilz (2021) find that languages with more speakers tend to have larger phoneme inventories, shorter words in number of syllables and a higher number of words per clause, among other things^4^. It therefore does not necessarily follow that changes in language structure should be attributed solely to the pressure for communicative efficiency, i.e., the balance between robust information transfer and articulation and processing costs, which rational language users try to achieve.

It is also important to keep in mind that transfer of information between the speaker and the addressee takes place in a noisy channel (Shannon, 1948; Gibson et al., 2019). This means that a message from Speaker to Addressee can be corrupted on the way – due to external noise, or due to production and processing errors. Therefore, there is a possibility that not all cues to a particular meaning or function are recovered from the signal. Producing only one cue to express a certain meaning may not be enough. In fact, typologists find redundancy at all linguistic levels (Hengeveld and Leufkens, 2018).

It is not surprising then that not all potential trade-offs are detected in actual linguistic data. For example, Sinnemäki (2008) finds significant inverse correlations between rigid word order and the presence of case marking of the core arguments in a representative sample of languages (also see below), but no correlation between word order and verb agreement, or verb agreement and case marking. Moreover, different cues may work in synergy. As an illustration, consider verbal and visual cues in communication. One would believe that processing one modality should be at the cost of the other. However, Holler et al. (2018) demonstrate that interlocutors respond faster to questions that have an accompanying manual and/or head gesture, than to questions without such visual components. According to Holler and Levinson (2019), multimodal information is easier to process than unimodal information (at least, for neurotypical speakers) thanks to synergy effects and creation of Gestalts.

To summarize, trade-offs, or inverse correlations, between linguistic variables related to different cues do not automatically imply efficiency as a driving force of language use and change, and the other way round.

I will illustrate these considerations by a case study of linguistic cues that help language users understand “who did what to whom.” There are multiple cues that help to infer this information: case marking, verb agreement, word order, and semantics. Languages differ in how they employ these cues. For example, Hungarian has case marking, agreement, but flexible word order (Pleh and MacWhinney, 1997), while others rely mostly on rigid word order, such as Present-Day English or Mandarin Chinese.

In this article, I will focus on four types of cues, which will be obtained from corpora in thirty languages, annotated with the Universal Dependencies (Zeman et al., 2020). The cues are as follows:

• Case marking, measured as Mutual Information between grammatical role and case;
• Semantic tightness, measured as Mutual Information between role and lexeme (lemma);
• Rigid word order, measured as 1 minus entropy of Subject and Object order;
• The proportion of clauses with verb-middle order, which is claimed to facilitate processing in a noisy channel (Gibson et al., 2013).

The role of these cues is discussed in section “Cues for Identification of Subject and Object.” The previous studies of these cues in typology focused mostly on binary trade-offs, such as rigid word order vs. case marking (Sinnemäki, 2014b), and case morphology vs. verb-medial order (Sinnemäki, 2010). Other cues and their relationships have received less attention, however, the present study is the first attempt to examine all four cues systematically with the help of quantitative measures and corpus data, which are presented in section “Data and Variables.”

Using pairwise correlations, I will show that the relationships are quite complex (see section “A Correlational Analysis of Cross-Linguistic Data”). Not all these cues are correlated, and not all correlations are negative. There is a robust negative correlation, however, between rigid word order and case marking. Next, I will move from binary correlations to causal networks in section “A Causal Analysis of Subject and Object Cues” (cf. Blasi and Roberts, 2017). Causal networks are more informative, because they allow us to identify directional relationships between different variables. There are some studies that employ diverse types of causal inference for different types of linguistic questions (e.g., Moscoso del Prado Martín, 2014; Baayen et al., 2016; Blasi, 2018; Dellert, 2019), but the approach has not yet become mainstream. In this article, I explore how causal inference based on synchronic corpus data can be used in token-based functional typology (Levshina, 2019). This type of corpus-based approach complements recent miniature language learning experiments that investigate the links between communicative efficiency (and other learning biases) and different linguistic cues to the same linguistic meaning (e.g., Culbertson et al., 2012; Fedzechkina et al., 2016; Kanwal et al., 2017; Kurumada and Grimm, 2019; Fedzechkina and Jaeger, 2020, to name just a few). Corpora are a valuable source because they represent language produced in naturalistic settings by real language users. I will demonstrate that some of the corpus-based results converge with previous experimental results (in particular, Fedzechkina et al., 2016; Fedzechkina and Jaeger, 2020), which shows that causal analysis can be added as a useful tool for studying linguistic cues across languages. I interpret the resulting causal network, discussing a possible diachronic scenario, which involves extralinguistic factors, such as the number of adult L2 learners. I argue that the potential for efficient and rational behavior playing a role in this scenario is quite limited.

## Cues for Identification of Subject and Object

### Formal Marking

This section describes different cues which can help to communicate “who did what to whom.” One type of cues is formal marking, most importantly, case marking and agreement (indexing). Some languages have consistent case marking on either the subject, the object, or both. For example, Lithuanian nouns, with the exception of some loan words, have distinct nominative and accusative case forms in all declension types. Some languages have differential marking, when A or P are marked in some situations, and not marked in others. For example, in Spanish, only animate and specific objects are marked, while other objects are unmarked (see more examples in Aissen, 2003). There are also case systems in which the distinctions between the Nominative and the Accusative forms are made only in some lexical classes, while the forms are identical in others, e.g., inanimate masculine nouns in Russian, e.g., *stol*-Ø “table.NOM/ACC”, or neuter nouns in Latin, e.g., *bell-um* “war-NOM/ACC”.

In some languages, the marking is probabilistic. An example is Korean (Lee, 2009), where the object markers are more or less likely depending on animacy, definiteness, person, heaviness of the object and other factors. Often, variation is contextual. For example, the Japanese object marker is used more frequently when the role configurations are not typical, e.g., when it is a thief who arrests a policeman, and not the other way round (Kurumada and Jaeger, 2015).

Both in probabilistic and categorical differential marking systems, there is a negative correlation between the presence of the case marker and predictability or accessibility of the role given the semantic and other properties of the nominal phrase. This correlation can be explained by efficiency considerations and rational behavior (e.g., Jäger, 2007; Levshina, 2021).

The arguments can also be marked on the verb. This is called agreement, or indexing. Subject indexing is popular across languages, e.g., German *er komm-****t*** “he comes”. As for object indexing, it is less frequently obligatory. The reason is that the relevant grammatical elements usually do not advance further down the cline of grammaticalization and do not become obligatory agreement markers, as it very often happens with subject agreement. Typically, object markers remain at the stage of differential object indexing (Haig, 2018). Their use or omission depends on diverse semantic and pragmatic factors, which are similar to the ones relevant for differential case marking. For example, in Maltese, the index is always present if the object is pronominal and given, and is always absent if it is new and non-specific. In the remaining situations, there is variation (Just and Čéplö, in press)^5^. This means that the use of differential object indexing is efficient.

### Word Order Cues

Fixed word order can also help the addressee to understand who did what to whom. It is used as a compensatory strategy in languages without case marking (Sapir, 1921). The position of the verb can be another cue. It is claimed that it is easier to assign the roles when the verb occurs between the subject and the object:

*[V]erb position is the particular vehicle which most conveniently enables these basic grammatical relations to be expressed by means of word order: the subject occurs to the immediate left, and the object to the immediate right of the verb. I.e., the verb acts as an anchor (Hawkins, 1986, 48–49).*

In experiments that involve gestural communication, participants prefer SOV when trying to convey a transitive event (Goldin-Meadow et al., 2008; Gibson et al., 2013; Hall et al., 2013). However, when an event is reversible, i.e., both participants can be Subject or Object, such as “The mother hugs the boy” and “The boy hugs the mother”, users tend to use SVO more often than when the role assignment is clear (Hall et al., 2013). Notably, some participants in Gibson et al. (2013) used some sort of *ad hoc* “spatial marking” that helps to distinguish between Subject and Object. For example, they used one hand to designate Subject and the other to represent Object, or gestured Subject in one location in space and Object in another. In the presence of such marking, they used the SVO order less frequently. Thus, SVO is used more often in the absence of any – formal or semantic – cues.

How can one explain these findings? Gibson et al. (2013) argue that verb-medial order is more robust to the presence of noise as far as conveying the roles of subject and object are concerned. If the addressee fails to recognize one of the nouns before the verb, he or she will be unable to decide if the noun is a subject or an object. For example, if instead of *The mother the boy hugs*, he only hears, *The mother hugs*, it will be difficult to interpret the role of the argument in the absence of the second nominal phrase, if there are no other cues. But if one noun is before the verb and one is after the verb, then the noise is less disruptive. If the argument that the addressee discerns is before the verb, e.g., *The mother hugs*, it can be identified as the subject. If the noun is after the verb, e.g., *Hugs the boy*, then it should be the object.

At the same time, Hall et al. (2015) show that pantomime comprehenders interpret SOV sequences robustly as subject-first, for both reversible and non-reversible events. This means that the role of ambiguity avoidance is probably less important than previously assumed (cf. Wasow, 2015). It may be that the preference for SVO in production has to do with avoidance of two semantically similar elements in close proximity. In linguistics, one speaks of the *horror aequi* principle, which describes the tendency to avoid placing formally, structurally or semantically similar units close to one another (cf. Ferreira and Firato, 2002; Rohdenburg, 2003; Walter and Jaeger, 2008). In phonology, this constraint is known as the Obligatory Contour Principle (Leben, 1973). By using the SVO order, the signers may avoid interference based on semantic similarity of Subject and Object.

### Semantic and Pragmatic Properties of the Arguments

Semantics of the arguments can provide strong cues for assigning the roles. For example, one can expect that it is a dog who bites a man, a hunter who kills a bear, a journalist who interviews a politician, and not the other way round.

There are also strong associations between roles and more abstract referential features, such as animacy, definiteness, discourse status, etc. According to cross-linguistic spoken corpus data, if an argument is human, 1st or 2nd person, definite or discourse-given, it is more likely to be Subject than Object. If an argument is non-human, 3rd person, indefinite or new, it is more likely to be Object than Subject.

Languages differ in how flexible they are with restrictions in the expression of Subject and Object. For example, Lummi (Straits Salish, British Columbia) does not allow the person of the subject argument to be lower on the person scale than the person of a non-subject argument. For example, if the subject in a potential active sentence is 3rd person and the object is 1st or 2nd person, then passivization is obligatory. In English, active sentences of this kind are possible, although there is a tendency to use passive more often in those cases (Bresnan et al., 2001).

A comparison of the associations between grammatical roles and semantics in English and German was performed by Hawkins (1986: 121–127, 1995) and extended cross-linguistically by Müller-Gotama (1994). For instance, Present-Day English has fewer semantic restrictions on the subject and object than Old English or German. Consider several examples below.

(1) a. Locative: *This tent sleeps four.*
      b. Temporal: *2020 witnessed a spread of the highly infectious coronavirus disease.*
      c. Source: *The roof leaks water.*

This suggests that subjects in English are less semantically restricted than subjects in German and Russian, in which these sentences would sound unnatural or incorrect (see also Plank, 1984). We can also say that English is a “loose-fit” language, while German, as well as Russian, Korean and Turkish, are “tight-fit” languages. A corpus-based study of thirty languages showed that the tightness rankings can be reproduced with the help of Mutual Information between grammatical roles and lexemes (Levshina, 2020b) – a method also used in the present article.

### Correlations and Causal Links From Previous Studies

Some correlations between the variables are already known from the previous studies. In particular, there is an inverse correlation between argument marking and rigid word order (Sapir, 1921; Sinnemäki, 2014b). Also, Greenberg’s (1966b) Universal 41 says: “If in a language the verb follows both the nominal subject and nominal object as the dominant order, the language almost always has a case system.” This means that verb-final order is associated with case marking, while verb-medial order is associated with lack of case marking (Sinnemäki, 2010).

Hawkins (1986) wrote about a positive correlation between verb-finalness and semantic tightness, which has been confirmed empirically (Levshina, 2020b). Moreover, he predicted a positive correlation between case marking and semantic tightness. Verb-final languages should be semantically tight and have case marking because an early incorrect assignment of roles would result in re-analysis, which has high cognitive costs.

As for the causal relationships, we know much less. Some diachronic accounts suggest that word order can determine case marking, according to the principle *post hoc ergo propter hoc*. According to Kiparsky (1996), the shift to VO began in Old English. It happened before the case system collapsed, and also before the loss of subject-verb agreement. Bauer (2009) demonstrates that that the change to VO and rigid word order in Late and Vulgar Latin was before the loss of inflection, which happened later in Romance.

There is also some support of this hypothesis in experimental linguistics. Fedzechkina et al. (2016) had their participants learn a miniature artificial language. The languages contained optional case marking on the object. Some languages had fixed constituent order, and some had flexible order. Learners of the fixed order language produced case marking significantly less often than learners of the flexible order language. In addition, a follow-up study by Fedzechkina and Jaeger (2020) demonstrates that the loss of marking in a fixed-order artificial language is observed only when case production requires additional effort, which indicates that the learners’ behavior is motivated by communicative efficiency and not by other considerations.

In the study presented below, I will investigate the correlational and causal relationships between four variables: case marking, rigid word order, verb-medial order and semantic tightness. These variables will be estimated with the help of corpus data, which are described below.

## Data and Variables

### Corpus Data

Available cross-linguistic syntactically annotated collections, such as the Universal Dependencies corpora (Zeman et al., 2020), are too small for the purposes of the present study because one cue type, namely, semantic tightness, requires distributional information about the frequencies of individual lexemes as Subject and Object. This is why I used freely downloadable web-based corpora from the Leipzig Corpora Collection (Goldhahn et al., 2012). These corpora contain collections of randomized sentences in diverse languages. The language sample consists of thirty languages (see Table 1). For each language, I took one million sentences representing online news (categories “news” and “newscrawl”). The choice of languages and the sample size were determined by the availability of language models in the UDPipe annotation toolkit, which was used to tokenize, lemmatize and annotate the sentences morphologically and syntactically (Straka and Straková, 2017). The processing was performed with the help of the R package *udpipe* (Wijffels, 2020). Importantly, the models provide uniform parts-of-speech tags and dependency relations (Universal Dependencies), which allows us to compare the data in different languages.

**TABLE 1:** Languages in this study.

**Language**	**Genus**	**Family**	**UD model**
Arabic	Semitic	Afro-Asiatic	arabic-padt-ud-2.4
Bulgarian	Slavic	Indo-European	bulgarian-btb-ud-2.4
Croatian	Slavic	Indo-European	croatian-set-ud-2.4
Czech	Slavic	Indo-European	czech-pdt-ud-2.4
Danish	Germanic	Indo-European	danish-ddt-ud-2.4
Dutch	Germanic	Indo-European	dutch-alpino-ud-2.4
English	Germanic	Indo-European	english-ewt-ud-2.4
Estonian	Finnic	Uralic	estonian-edt-ud-2.4
Finnish	Finnic	Uralic	finnish-tdt-ud-2.4
French	Romance	Indo-European	french-gsd-ud-2.4
German	Germanic	Indo-European	german-gsd-ud-2.4
Greek (modern)	Greek	Indo-European	greek-gdt-ud-2.4
Hindi	Indic	Indo-European	hindi-hdtb-ud-2.4
Hungarian	Ugric	Uralic	hungarian-szeged-ud-2.4
Indonesian	Malayo-Sumbawan	Austronesian	indonesian-gsd-ud-2.4
Italian	Romance	Indo-European	italian-isdt-ud-2.4
Japanese	Japanese	Japanese	japanese-gsd-ud-2.4
Korean	Korean	Korean	korean-gsd-ud-2.4
Latvian	Baltic	Indo-European	latvian-lvtb-ud-2.4
Lithuanian	Baltic	Indo-European	lithuanian-hse-ud-2.4
Persian	Iranian	Indo-European	persian-seraji-ud-2.4
Portuguese	Romance	Indo-European	portuguese-bosque-ud-2.4
Romanian	Romance	Indo-European	romanian-rrt-ud-2.4
Russian	Slavic	Indo-European	russian-syntagrus-ud-2.4
Slovenian	Slavic	Indo-European	slovenian-ssj-ud-2.4
Spanish	Romance	Indo-European	spanish-gsd-ud-2.4
Swedish	Germanic	Indo-European	swedish-talbanken-ud-2.4
Tamil	Southern Dravidian	Dravidian	tamil-ttb-ud-2.4
Turkish	Turkic	Altaic	turkish-imst-ud-2.4
Vietnamese	Viet-Muong	Austro-Asiatic	vietnamese-vtb-ud-2.4

This annotation was used to extract all nominal subjects and objects. Here and below by subjects I mean only subjects of transitive clauses. Intransitive clauses were not taken into account. Pronominal arguments were excluded for the sake of comparability. Some languages are pro-drop, and it would be technically impossible and linguistically incorrect to recover the “missing” pronouns.

Of course, using automatic annotation is risky. Additional checks were performed in order to make sure that the subjects and objects are identified correctly. Moreover, another study (Levshina, 2020a) compared several word order and case marking scores based on the online news corpora and the training corpora in the UD collection. It revealed very strong positive correlations between the scores based on these two data sources, which can serve as an indication that the data are reliable.

### Variables

#### Case Marking

Case marking is represented here as Mutual Information between Role (Subject or Object) and Case (depending on the case inventory in a particular language). In comparison with traditional classifications, such as the number of morphological cases in a language, this method can determine more precisely the amount of information obtained about Role through observing Case in language use. This is particularly important for languages with differential case marking. For example, in Russian some nouns have different forms in the Nominative and Accusative (e.g., *devočk-a* “girl-NOM” and *devočk-u* “girl-ACC”), while some nouns have identical forms (e.g., *stol* “table” or *myš* “mouse”). Similarly, as already mentioned, Korean has variable marking on Subject and Object with complex probabilistic rules (Lee, 2009). In some languages, like Finnish and Estonian, the same morphological cases (e.g., Nominative and Partitive) can express both Subject and Object under certain conditions. The question is then, how frequently do the Subject and Object forms help the addressee to infer the grammatical role of a noun? In order to answer this question, we need a quantitative corpus-based approach.

The frequencies of Role-Case combinations were determined in the following way. In some languages, the roles are marked by adpositions or case particles marking the roles that are treated as separate words by the Universal Dependencies, e.g., the preposition *a* in Spanish. In this case, I simply counted the number of Subjects and Objects with and without these markers, which are marked with the dependency “case.” Table 2 displays the counts for Spanish.

**TABLE 2:** Frequencies of case forms in Spanish.

**Case**	**Subject**	**Object**
Zero marking	126,736	569,252
Preposition *a*	0	55,442

If a language has a special Subject form, which cannot be used to represent Object, I counted in three Cases (rows in the table): strictly the Subject form, the Object form and the ambiguous form, which usually has zero marking. For example, Hindi has three Cases under this approach: absolutive (with zero marking), ergative (only transitive subjects) and accusative (only transitive objects). Table 3 represents the counts for Hindi. A similar situation is in Japanese and Korean, which have Subject-only particles, Object-only particles, and unmarked forms.

**TABLE 3:** Frequencies of case forms in Hindi.

**Case**	**Subject**	**Object**
Absolutive (zero marking)	46,241	363,647
Ergative	61,512	0
Accusative	0	92,510

In order to obtain the counts of morphological cases, I used two approaches: automatic and manual. The automatic method was used in simple case systems. I compared the case wordforms with the corresponding lemmas, which represent the Nominative (Subject) case. This is how I obtained the counts for Object forms in several languages. In more complex situations, I analyzed manually samples of 200 Subjects or Objects (or 500, if the system was relatively simple to analyze) with the help of dictionaries, and obtained the counts by extrapolating the frequencies from the sample. This procedure was used in those languages in which automatic comparison of case wordforms with lemmas was problematic because of the presence of other morphemes, e.g., definite articles or possessive suffixes, as in Arabic, Bulgarian, Finnish or Hungarian. Table 4 displays the extrapolated counts for Finnish. It has Nominative (no marking), Genitive and Partitive cases that are used with Subject and Object. Subjects can be expressed by the zero Nominative and occasionally by Partitive and Genitive forms, while Objects can have no marking, or be in the Partitive or Genitive form with case suffixes.

**TABLE 4:** Frequencies of case forms in Finnish (extrapolated).

**Case**	**Subject**	**Object**
Nominative (zero marking)	132,631	94,077
Genitive + Partitive	9,562	386,268

Note that in order to perform the automatic comparison and facilitate the manual annotation, I took only non-plural and non-dual forms in all languages, so that the formal variation based on number could be excluded. I do not expect this restriction to influence the results strongly because plural forms are less frequent than singular ones (Greenberg, 1966a).

German was treated in a special way because the carriers of case information are the articles, pronouns and adjectives, e.g., the nominative form *der Tisch* “the table” is contrasted with the accusative form *den Tisch*. This contrast is only available for masculine nouns. I inferred the number of marked forms by computing the number of masculine singular nouns in the role of Subject and Object, which are modified by determiners or adjectives. Feminine and neuter nouns, as well as the masculine ones without determiners or adjectives, were treated as having ambiguous forms.

Next, for each Case-by-Role frequency table, I computed Mutual Information (MI) between Case and Role:

$$I\left(Case;Role\right)=\sum_{i,j}p\left(case_{i},role_{j}\right)log_{2}\frac{p\left(case_{i},role_{j}\right)}{p\left(case_{i}\right)p\left(role_{j}\right)}$$

Finally, in languages without any Subject or Object markers (that is, Danish, Dutch, English, Indonesian, Swedish, and Vietnamese), the MI scores were set to 0. Note that in some case-free languages, e.g., in French, a tiny fraction of objects are marked with a preposition. These are objects representing unspecified quantity, e.g., *Je voudrais de l’eau* “I would like some water.”

**FIGURE 1:**
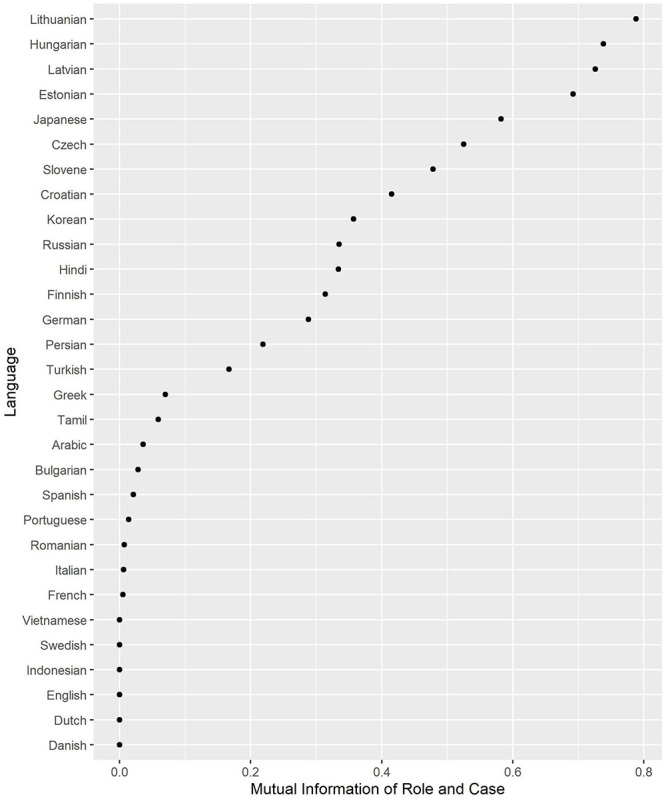
Case marking (Mutual Information between Role and Case).

The MI scores are displayed in Figure 1. The languages at the bottom have no or very limited case marking (English, Indonesian, the Romance languages and Vietnamese), while the languages at the top have extensive marking, which contributes substantially to discriminating between Subject and Object (e.g., the Baltic languages and Hungarian). Lithuanian, the Indo-European language that has preserved most of the ancient nominal morphology, has the highest distinctiveness. Most Slavic languages, Hindi, Persian, and Turkish and other languages with differential marking are in the middle, as expected. The low score of Spanish, which has differential object marking, as well, is somewhat surprising. The reason may be that animate specific objects, which are marked with the preposition a, are much rarer than other nominal phrases (see Table 2).

Agreement markers are not investigated in this article. There are several reasons. First, it is difficult to quantify how much they help to distinguish between Subject and Object. Second, previous research has shown that subject agreement is not significantly correlated with other cues, such as word order or case marking (Sinnemäki, 2008). At the same time, it has been found that object agreement is not observed when both other cues are present simultaneously in a language. At the moment, my sample of languages does not allow me to test the role of object agreement statistically. I leave that to future research.

#### Semantic Tightness

**FIGURE 2:**
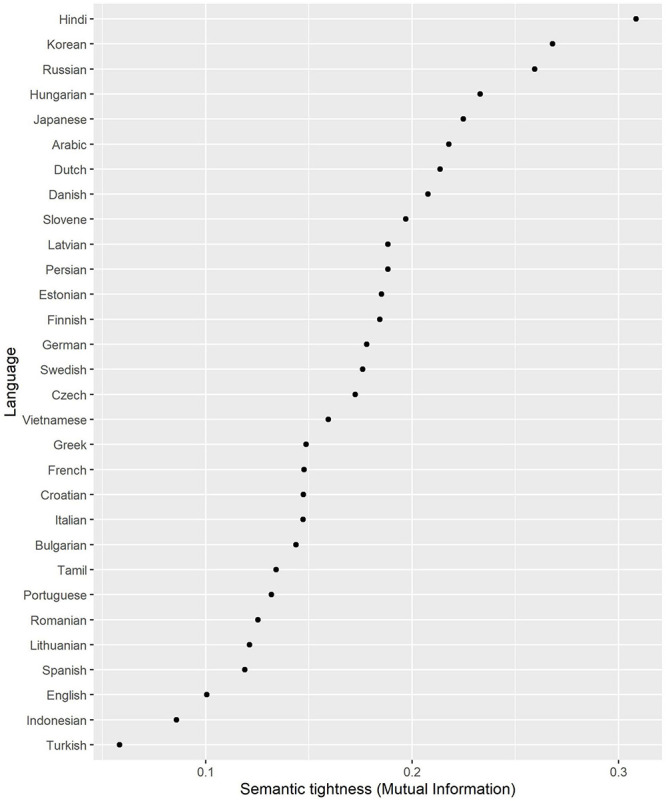
Semantic tightness (Mutual Information between Role and Lexeme).

As a proxy for semantic tightness, I computed Mutual Information between Role and individual lexemes. For this purpose, I extracted frequencies of common nouns as Subject and Object from the corpora. Examples are displayed in Table 5. Usually, human nouns tend to be biased toward the role of Subject (e.g., *hunter*), while inanimate nouns more frequently occur in the object role (e.g., *t-shirt* and *street*). The stronger these biases, the higher the MI score and therefore the tighter the semantic fit. The MI scores are shown in Figure 2.

**TABLE 5:** A fragment of the Lexeme – Role matrix for English.

**Lexeme (lemma)**	**Transitive subject**	**Object**
hunter	40	22
street	34	466
t-shirt	3	118

The tightest languages are Hindi, Korean, Russian, Hungarian, and Japanese. This supports previous accounts (see section “Semantic and Pragmatic Properties of the Arguments”). Among the loosest languages are English and Indonesian, which are also well known as semantically loose. It is surprising that Turkish is the loosest language in the sample, although if we also take into account more grammatical roles (such as intransitive subjects and obliques), it becomes relatively tight (Levshina, 2020b).

An important issue in language comparison is what to count as a word (Haspelmath, 2011). For example, in English, the phrase *art history* consists of two words, but its German equivalent *Kunstgeschichte* is only one word. In order to counterbalance the influence of orthographic conventions, I also computed the scores treating multiword units like *art history* as one lexeme, based on the Universal Dependencies “compound”, “fixed” and “flat”. In the subsequent correlational and causal analyses, this variable, however, did not perform differently from the first one. This is why the analyses presented below are based only on lemmas of single orthographic words (but see Levshina, 2020b).

#### Rigid Word Order

The next type of information reflects if rigid word order can be a reliable cue of the syntactic roles. In order to compute it, I used anti-entropy, which is 1 minus Shannon entropy of the order of Subject and Object. The formula for computing entropy of orders SO and OS is as follows:

$$H=-\sum_{i=1}^{n}P\left(Order_{i}\right)*logP\left(Order_{i}\right)$$

where P (Order_*i*_) stands for the probability of SO or OS. The probabilities were computed as simple proportions of each word order in the corpora. More on this approach can be found in Levshina (2019).

**FIGURE 3:**
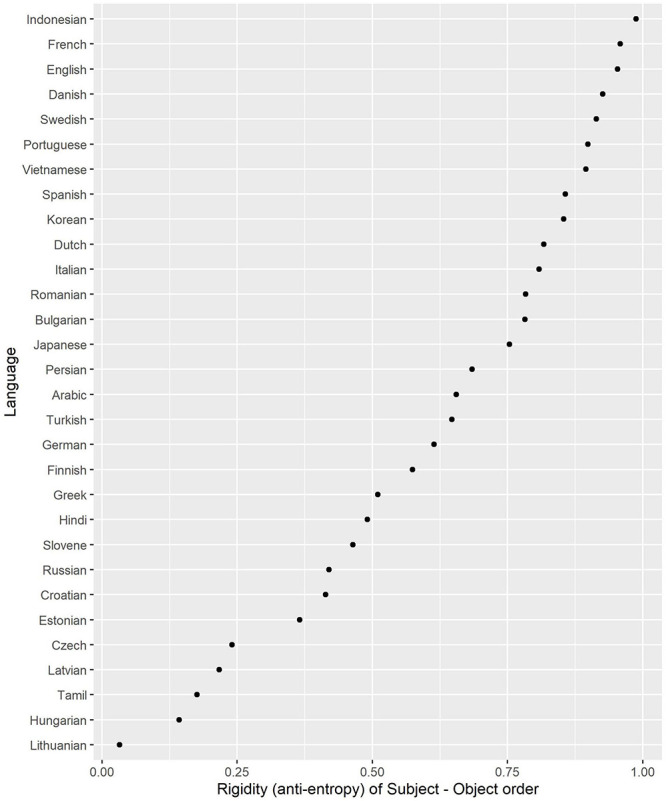
Rigidity of Subject – Object order (1 – entropy).

If either Subject is always before Object or the other way round, i.e., P (SO) = 1 and P (OS) = 0, or P (SO) = 0 and P (OS) = 1, the entropy value is minimal (H = 0) and therefore the rigidity score is maximal: 1 – H = 1 – 0 = 1. If both orders have equal probabilities, i.e., P (SO) = P (OS) = 0.5, then the entropy value is maximal (H = 1) and the rigidity score is minimal: 1 – 1 = 0. The rigidity scores are displayed in Figure 3.

The Baltic, Finno-Ugric and most Slavic languages, as expected, have the lowest rigidity scores, allowing for word order flexibility. In contrast, English, French, Indonesian have the most rigid order, followed by the Scandinavian and other Romance languages and Vietnamese. Interestingly, Korean and Japanese do not display much variability, although it is assumed that they have flexible order of Subject and Object.

#### Verb-Medial Order

**FIGURE 4:**
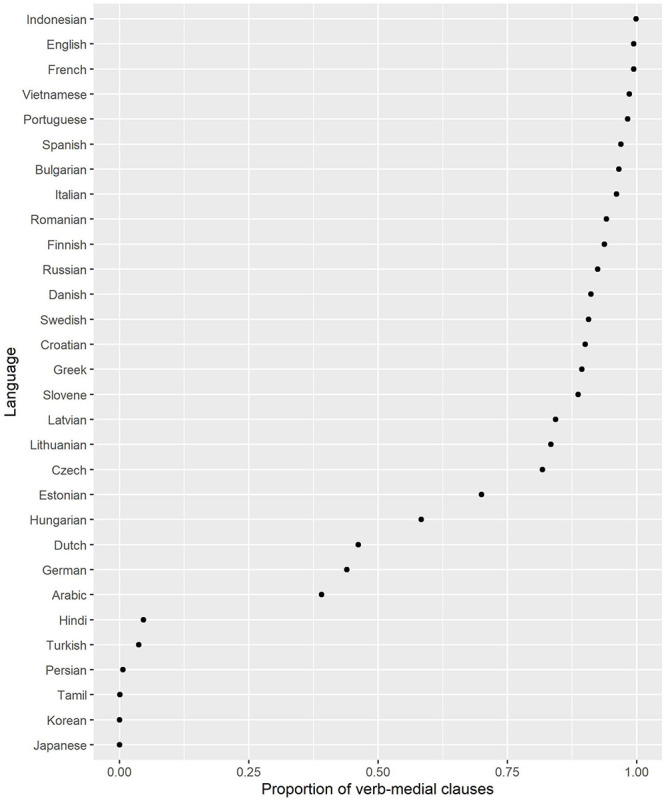
Proportion of verb-medial clauses.

The fourth and final variable considered in this study is “verb-medialness,” which shows how frequently the head verb occurs between the subject and the object. The procedure was as follows. I computed the number of clauses in the corpora (only finite main and subordinate clauses with a lexical verbal predicate were considered), which had overt Subject and Object, and a lexical head verb. Next, I computed the proportion of all clauses where the verb is between Subject and Object (in either order). The scores based on the UD corpora and the online news corpora are displayed in Figure 4. One can see a gap between the typical SOV languages (Japanese, Tamil, Korean, Hindi, and Turkish) with the lowest scores and all the rest. Indonesian, English and French are nearly always verb-medial.

## A Correlational Analysis of Cross-Linguistic Data

### The Problem of Dependent Observations

Computing correlations between the variables in this case study is not straightforward because the dataset contains dependent observations. Many languages come from the same family or even genus. In order to address this issue, I used a combination of sampling and permutation. I followed Dryer’s (1992) approach relying on genera as the main taxonomic level. In 1,000 simulations, I sampled only one language from each genus and computed the Spearman’s rank-based correlation coefficients for each sample. These coefficients were then averaged for each pair of variables. The Spearman method was used because some of the relationships displayed small non-linearity, but Pearson’s product-moment coefficients, as well as Kendall’s coefficients, reveal similar results.

In order to perform the null hypothesis significance testing, I computed and logged the test statistic for the original pairs of scores in every simulation. I also ran 1,000 permutations, in which the original scores of the second variable were randomly reshuffled. The permutation scores represented the distribution of the test statistic under the null hypothesis. Next, I counted the number of cases out of 1,000 permutations where the permuted scores were equal to or more extreme than the original test statistics based on the unpermuted data. These proportions served as *p*-values. The *p*-values were then averaged across the 1,000 samplings from the genera.

### Results of Correlational Analyses

**FIGURE 5:**
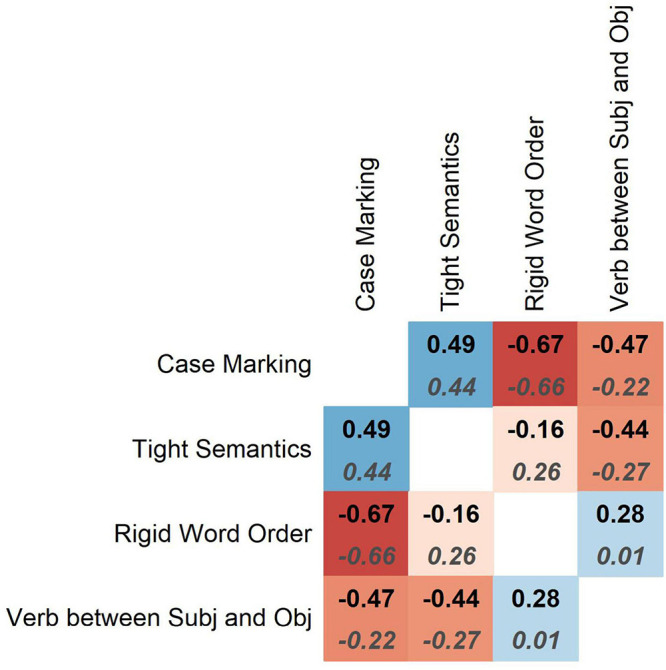
Spearman’s correlation coefficients between pairs of variables, averaged across 1,000 simulations. Top: simple pairwise coefficients. Bottom: partial coefficients.

The Spearman correlation coefficients are displayed in Figure 5. The 95% confidence intervals around the average values can be found in Appendix Table 1. The simple (non-partial) pairwise correlations are represented by bold labels at the top of the squares. The strongest negative correlation is between case marking and rigid order of Subject and Object. The correlation is negative and significant (*ρ* = −0.67, *p* = 0.004). This means that distinctiveness of case marking increases with word order flexibility and decreases with word order rigidity. Next follows a positive correlation between case marking and tight semantics (*ρ* = 0.49, *p* = 0.043). From this we can conclude that semantically tight languages tend to have more informative case marking than semantically loose ones. The negative correlation between case marking and proportion of verbs located medially, between Subject and Object (*ρ* = −0.47, *p* = 0.042), means that languages without distinctive case marking tend to have SVO. There is also a negative correlation between semantic tightness and the proportion of verbs in the middle (*ρ* = −0.44, *p* = 0.047). This suggests that semantically loose languages are usually verb-medial, whereas semantically tight ones are usually verb-final (the only language in the sample with partly verb-initial order is Arabic). The remaining correlations are not significant.

If we compute partial correlations, which represent the relationships between variables X and Y taking into account all other variables, as in multiple regression, the direction of the significant correlations is mostly similar, as one can see from the coefficients represented by dark-gray labels in italics in Figure 5. The 95% confidence intervals around the average coefficients can be found in Appendix Table 1. The correlations between rigid order and case marking, and between tight semantics and case marking change very little, but the correlations between the proportions of verbs in the middle and the other variables become much weaker. In this case, only the correlation between rigid word order and case marking is statistically significant at the level of 0.05 (*p* = 0.012).

To summarize, we see that not all correlations are negative (though all significant partial correlations are): the correlation between semantic tightness and case marking is positive, for example. Also, not all variables are correlated (although this can be due to the relatively small sample size). It is also remarkable that case marking is the most strongly correlated with the other variables.

## A Causal Analysis of Subject and Object Cues

### Motivation for Causal Analysis

Hypotheses about causal mechanisms can be performed with the help of experiments, by manipulating the variables of interest while carefully controlling for possible confounding effects. If diachronic data are available, causal relationships can be discovered with the help of a Granger-causality analysis (Moscoso del Prado Martín, 2014). Here I will use statistical methods to identify causal relationships using the synchronic observational data. In this case, causal analysis is based on tests of conditional independence of one variable X from another variable Y, given another variable (Z) or variables. Independence between X and Y means, informally speaking, that we do not know more about the value of X if we know the value of Y, and the other way round. For example, if we know that it will rain today, this information will not help us to guess the exchange rate of euro to British pound sterling. Conditional independence means that we cannot say anything more about X if we know Y, given Z. For example, if we take children’s heights and their vocabulary size, we are likely to find a positive correlation. But if we control for age, this correlation will disappear. In this case, there are several scenarios of causal relationships. For example, the relationship between X and Y can be a so-called fork X ← Z → Y, which means that Z is the common cause for both X and Y. This can be illustrated by the above-mentioned example with age as the common cause of height and vocabulary size. A linguistic example is lexical borrowing into different languages from English. If we take two unrelated languages, e.g., Japanese and Telugu, and compare their vocabularies, we will find that they overlap to some extent due to shared loanwords. But if we control for English loans, the languages will become independent (Dellert, 2019: 69). This is not the only possibility when X and Y are conditionally independent given Z. The relationships can also represent a causal chain, X → Z → Y or X ← Z ← Y, where all the influence from X to Y or from Y to X is mediated by Z. For example, there is a dependency between Modern English and Old English, but it is mediated by Middle English. More variables are needed in order to distinguish between forks and different kinds of chains.

Consider now the opposite scenario: X and Y are independent in the absence of Z, but become dependent if we control for Z. In this case, the variables are likely to form a so-called collider, or v-structure: X → Z ← Y. To give a very basic example, we can assume that the amount of talent (X) and amount of luck (Y) are independent. We can also assume that they both contribute to success (Z). If we control for success, talent and luck will become dependent. That is, if we know how successful one is, and the amount of talent, we can figure out the amount of luck. For instance, if someone has achieved a lot, but has no talent, people will say that he or she has been very lucky. And if someone is obviously talented, but remains an underdog, then bad luck is to blame.

There are many different algorithms for causal inference. Here I use the FCI (Fast Causal Inference) algorithm, which is preferred in the situations when we are not sure if the assumption of causal sufficiency is met. This means that we could miss some other variables that represent common causes for two or more variables in the data (Spirtes et al., 2000; Dellert, 2019: 80). In other words, FCI allows latent variables. In our case, potential latent variables can be sociolinguistic ones, such as intensity of language contact or population size (e.g., Trudgill, 2011; see also section “A Possible Diachronic Scenario”). The relevance of different sociolinguistic variables for grammar, however, is not fully understood yet (Sinnemäki and Di Garbo, 2018).

FCI also allows unmeasured selection variables, which determine whether or not a measured unit (here: a language) is included in the data sample. They represent selection bias. In our case, this can be the fact that all languages in the sample are written languages with a large number of speakers. Also, these languages are spoken in Eurasia only.

The result of a FCI algorithm is a Partial Ancestral Graph (PAG), where causal relationships are represented as edges between nodes (here: linguistic cues). Different types of edges are possible. When a relationship is directional, it is represented as an arrow: X → Y. If variables X and Y have a common latent cause, the edge will be bidirectional: X ↔ Y. Undirected edges (X – Y) suggest the presence of selection variables. In addition, there can be edges X °→ Y, X °– Y and X °–°Y, where the circle represents uncertainty: it stands for either an arrowhead, or a tail.

The FCI algorithm runs as follows. The first step is to identify the undirected complete graph, or the skeleton. The algorithm used here is stable in the sense that the result does not depend on the order of variables in the data, cf. Colombo and Maathuis (2014). All edges of this skeleton are of the form X °–°Y. This means that they are undetermined, or not oriented. Next, v-structures are identified using conditional independence tests, and superfluous edges are removed if a conditional independence is found. Finally, the v-structures are oriented again, and all possible undetermined edge marks ° are eliminated using the orientation rules in Zhang (2008). See more details in Dellert, (2019: 80–85).

The causal analysis was performed with the help of the FCI algorithm implemented in the *pcalg* package in R (Kalish et al., 2012; R Core Team, 2020). The rank-transformed variables were used instead of the original ones, to ensure the compatibility with the correlational analyses.

Due to the presence of dependent observations the causal analysis was repeated 1,000 times on subsets of the data, where one language was picked randomly from every genus. In each iteration, the algorithm returned an asymmetric adjacency matrix with information about the edges from X to Y and from Y to X represented by number codes. The presence of every edge was tested with the significance level of 0.05. Every matrix was logged, and the different types of edges were counted and analyzed, as will be shown below.

### A Causal Network

**FIGURE 6:**
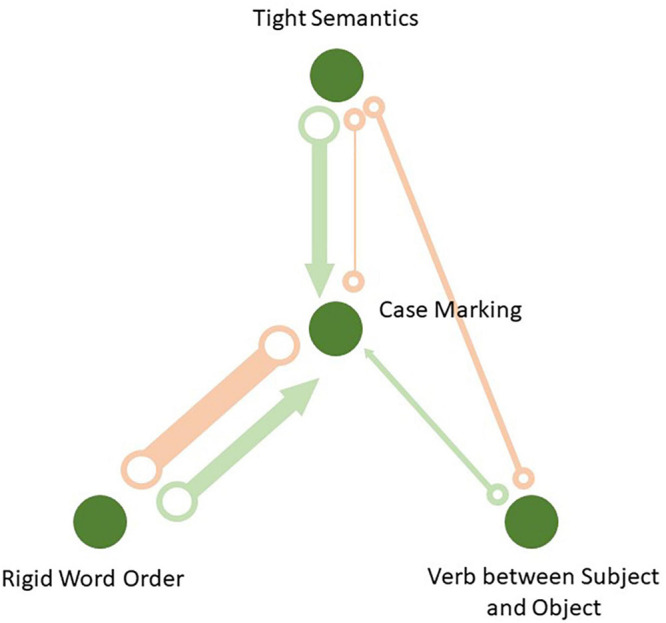
Causal network based on the FCI algorithm: Thickness of the edges reflects their frequencies in 1,000 samples.

The causal graph based on the FCI algorithm is displayed in Figure 6. The thickness of the edges corresponds to their frequency in 1,000 simulations, during which languages were randomly sampled from the genera. All links that have passed the significance test in at least one simulation are displayed in the causal network. One can see that some links are missing, which means the corresponding nodes are conditionally independent in all iterations at α = 0.05. In every simulation, the FCI algorithm computes maximal *p*-values for all conditional independence tests performed on every edge. If it is less than 0.05, the nodes are treated as conditionally dependent, and there exists a connection between them. The average *p*-values and their minimum and maximum values in the 1,000 simulations are displayed in Table 6.

**TABLE 6:** Mean *p*-values of the edges in FCI.

	**Case marking**	**Tight semantics**	**Rigid order**	**Verb middle**
Case marking		0.099 (0.002, 0.392)	0.011 (0.001, 0.068)	0.122 (0.027, 0.346)
Tight semantics	0.099 (0.002, 0.392)		0.564 (0.109, 1)	0.128 (0.021, 0.895)
Rigid order	0.011 (0.001, 0.068)	0.564 (0.109, 1)		0.319 (0.058, 0.750)
Verb middle	0.122 (0.027, 0.346)	0.128 (0.021, 0.895)	0.319 (0.058, 0.750)	

*In parentheses: minimum and maximum values.*

There are four edges which pass the conditional independence test at least once. The links are between case marking and word order, between case marking and verb-medialness, between case marking and semantic tightness, and between verb-medialness and semantic tightness. The causal network also represents two types of links which emerged during the simulation. Most links are so-called unoriented edges of the type X °–°Y, which means that no direction could be identified. Each end of such an edge could be an arrowhead or a tail. This can happen due to lack of v-structures, or colliders, in the sample. The most frequent link of this type is between rigid order and case marking. It occurred in 650 out of 1,000 iterations. Next comes the link between verb-medialness and semantic tightness with 59 occurrences. Finally, the link between tight semantics and case marking was observed only six times.

In addition, there were several partially directional edges of the type X °→Y. This means that there is no certainty whether the relationship is X →Y or it is bidirectional, X ↔Y. Recall that bidirectional edges suggest the presence of a common latent cause. Importantly, all of these edges have their arrowheads pointed to case marking. This means that case marking is more likely to be influenced by the other variables than the other way round. The most frequent edge of this type is the one from rigid word order to case marking with 344 occurrences in 1,000 simulations. It is followed by the edge from tight semantics to case marking with 314 occurrences, and finally by the link from verb-medialness to case marking, which occurred 30 times only. The edge between verb-medialness and semantic tightness does not have any partially directed links.

These results contain a lot of uncertainty. More data are apparently needed. Still, we can draw some conclusions. First of all, case marking is in the center of the graph. Second, we see that all partially directed edges lead to case marking, and none from case marking to the other cues. This suggests that formal marking is probably the most sensitive to other parameters’ influence.

Also, the total number of edges of any type between tight word order and case marking was 994 out of possible 1,000. It was present in almost all iterations. This means that the causal link between word order and case marking has by far the strongest support. However, we also see that there are some chances of a causal relationship from word order to case marking, and no partially or fully directed edges in the opposite direction.

The evidence for the link between tight semantics and case is weaker. The total number of edges between tight semantics and case marking was 320. Nearly all of them are partially directed. Therefore, a unidirectional effect of case marking on tight semantics is less likely than the reverse effect. There were 59 non-directed edges between tight semantics and verb-medialness. The total number of edges from verb-medialness to case marking was only 30, the smallest value. All these links were partially directed.

### A Possible Diachronic Scenario

How can we interpret these correlations and causal links? A tentative historical scenario could be as follows. Under normal circumstances, languages tend to accumulate complexity (Dahl, 2004), which explains why languages are vastly redundant (Hengeveld and Leufkens, 2018). Tight semantics and rich case morphology can be among those complexities. Mature and complex languages can also have complex contextual rules for choosing SO or OS for managing information flow, which makes the unconditional entropy of Subject and Object order high. All these complexities are not a problem for child L1 learners and are transmitted faithfully from one generation to another. Also, these languages can retain verb-final order, which was arguably the order in the ancestral language (Gell-Mann and Ruhlen, 2011).

Now imagine that due to increasing language contact the number of adult learners of this language increases. What would the consequences be like? We can expect the following changes.

First, evidence from artificial language learning experiments suggests that adults are better at learning word sequences that are produced by rules, while children are better at memorizing sequences without any underlying rules (Nowak and Baggio, 2017). Although there is evidence that adults tend to probability-match free variation in an input language under certain conditions more than children do (Hudson Kam and Newport, 2005)^6^, experiments with artificial languages show that input languages exhibiting free variation become increasingly regular, revealing a strong bias toward regularity in adult learners during language diffusion (Smith and Wonnacott, 2010). Moreover, it is important to emphasize that variation in word order is hardly ever free. On the contrary, it is constrained by individual constructions and stylistic and information-management considerations. It is then possible that a rigid order of Subject and Object, which represents a simple generalization, is easier for adult L2 learning than a so-called flexible order with many local rules^7^. Adults will learn patterns that can be captured by a few simple rules. As for L1 speakers in language contact situations, there is evidence that they prefer more rigid word order if they are immersed in another language. For example, Namboodiripad (2017) and Namboodiripad et al. (2019) show that increased language contact with English leads to a greater preference for canonical order (SOV) in Korean and Malayalam speakers. So we can expect the order to become more rigid in a language contact situation.

Second, the associations between roles and lexemes or semantic classes can become looser due to the cognitive limitations of adult learners. Acquisition of the role – semantics associations, and which constructions to use if some combinations are not allowed (e.g., passives), is difficult. Also, growth and increasing diversity of a language community can cause greater variability in the role – referent mappings^8^. Since L2 learners can subconsciously transfer their mother tongue features to the target language (Siegel, 2008: Ch. 5), this can increase the pool of variants in the expression of grammatical roles, which makes the associations between the roles and semantics looser.

Third, the verb can shift to the middle position due to increased noise in L2 communication. Following the hypothesis in Gibson et al. (2013), the verb-medial order is more robust for information transmission in a noisy channel. One can consider L2 communication noisier than L1 communication. In fact, if we look at high-contact pidgins and creoles represented in the Atlas of Pidgin and Creole Language Structures, we will find that 71 of 76 languages (93%) have SVO, with 63 languages (83%) relying on this word order as the exclusive or dominant pattern (Huber and the APiCS Consortium, 2013). According to Bentz and Christiansen (2010), the increase of L2 learners of (Vulgar) Latin as *lingua franca* of the expanding Roman Empire provided an important pressure toward the Romance SVO without case marking and the reduction of word order flexibility. It is also possible that the high proportion of L2 speakers is responsible for the predominant SVO in the three most widespread languages: Chinese, English, and Spanish. Bentz and Christiansen explain this development by production pressures. In particular, they claim that it is easier to assign the case to the object when the verb comes first.

We also see a weak causal link between the position of the verb and semantic tightness. According to Hawkins (1986), semantic tightness helps to avoid reanalysis in verb-final clauses and thus to avoid extra effort (see also Levshina, 2020b). This can be seen as a manifestation of efficiency. The model does not show which of the variables influences which one. It may be that tight semantics allows for verb-finalness, or verb-finalness leads to semantic tightness. More research is needed to understand this relationship.

Finally, case morphology represents another source of complexity, which L2 learners can be tempted to get rid of. In the causal network, we saw that there are some chances that the directional relationships are in fact bidirectional, which is usually due to latent common causes. It seems that the presence of L2 learners and similar sociolinguistic variables can be such a common cause.

In addition, the changes toward more rigid word order, semantic looseness and verb-medial order create favorable conditions for the language to lose case marking. Semantic looseness leads to more abstract semantics of the case forms, which do not contribute much beyond the syntactic relationships. Since the forms do not express much beyond what is already conveyed by word order, it would be rational and efficient to save articulatory and processing effort by not using case marking. The role of production effort in loss of case marking has been demonstrated in Fedzechkina and Jaeger’s (2020) experiment involving adult learners of an artificial language, so it is a valid factor. That said, it is important to emphasize that the loss of marking as a way of saving effort can happen only after appropriate conditions have been created.

## Conclusion

This case study investigated the relationships between different cues that help the addressee to assign the grammatical roles of Subject and Object in a transitive clause. The cues included case marking, tight association between lexemes and roles (semantic tightness), rigid order of Subject and Object, and the position of the verb between Subject and Object. The measures that reflect the prominence of these cues were obtained from corpora in thirty languages.

The results of the correlation analyses demonstrated that some cues were negatively correlated, and some were not. By far the strongest correlation is the inverse correlation between case marking and rigid order of Subject and Object. This correlation has been discussed in numerous previous accounts (e.g., Sapir, 1921; Sinnemäki, 2014b; Fedzechkina et al., 2016; Fedzechkina and Jaeger, 2020). Importantly, the correlation between word order rigidity and case marking distinctiveness is not influenced by the presence or absence of the other variables. Therefore, the relationship between word order and case marking is robust, which means that the previous studies that focused only on this pair of cues are valid.

The other correlations are also in accordance with the previous studies. Semantic tightness and case marking display a strong positive correlation: the more information is provided by the lexemes (semantics), the more distinctive are the case forms in a language. This supports Hawkins (1986) ideas about tight-fit and loose-fit languages, where semantic tightness is associated with case marking. The analysis also revealed an expected negative correlation between verb-medialness and semantic tightness (Hawkins, 1986; Levshina, 2020b). Moreover, languages with the verb between Subject and Object usually have no case marking (cf. Sinnemäki, 2010), and tend to have rigid word order. Verb-final languages can have flexible word order and usually have case marking. This ties in well with the results of the gesture experiment in Gibson et al. (2013), who found a correlation between verb-finalness and the use of spatial marking of the core arguments.

The results of the correlational analysis are in accordance with previous grammar-based and experimental studies, which means that corpus-based variables can be used successfully to represent the linguistic cues. At the same time, only rigid word order and case marking have a significant partial correlation when the other variables are taken into account. This finding requires further research on a larger sample of languages. Also, the results indicate that case marking is more strongly correlated with the other cues than any other variable – a fact that has not been previously reported.

The causal analysis based on the Fast Causal Inference algorithm showed that case marking is the variable that is the most likely to be affected by the other variables. The most probable causal link is found between rigid word order and case marking, with greater probability of the directional relationship from word order to case marking than the other way round. This supports the previous observations based on the history of English and the Romance languages (see section “Correlations and Causal Links From Previous Studies”), saying that fixation of word order and transition toward SVO triggered the loss of case marking. It also provides empirical evidence for the reasoning in Koplenig et al. (2017) about the directionality of this relationship. Importantly, it converges with the experimental results in Fedzechkina et al. (2016) and Fedzechkina and Jaeger (2020), which point in a similar direction. Also, cross-linguistic evidence (Sommer and Levshina, 2021) demonstrates that word order plays an important role in differential case marking of core arguments. The use of a case marker is more likely when the word order in a clause is different from the dominant one, supporting the experimental results in Fedzechkina and Jaeger (2020) and Tal et al. (2020). This effect is found in quite a few languages from all over the world, including Dazaga (Saharan), Gurindji Kriol (mixed), Kakua (Cacua-Nukak), Sheko (Afro-Asiatic), and Udihe (Altaic). Case markers are often used on topicalized objects in left dislocation (Iemmolo, 2010), but also in other situations. The function of case marking is to override the addressee’s expectations about the grammatical role of the argument and/or about the topic of the clause (cf. Diessel, 2019: Ch. 11).

At the same time, we do not find conclusive evidence that word order flexibility or rigidity is determined by the presence or absence of case. This goes against Sapir’s hypothesis, who wrote about the historical change in English, “[a]s the inflected forms of English became scantier, as the syntactic relations were more and more inadequately expressed by the forms of the words themselves, position in the sentence gradually took over functions originally foreign to it” (Sapir, 1921: 166). Although some languages are known to use word order freezing (i.e., choosing the dominant word order) in ambiguous contexts, in particular, when the case forms are not informative enough (Jakobson, 1971), this effect is relatively weak in real language use (see Berdicevskis and Piperski, 2020 on Russian and German), so it is unlikely to have a major impact on language change.

Moreover, the causal analysis shows some probability that case marking can be affected by semantic tightness. We also find some weak evidence that the position of the verb can affect case marking, as well. In addition, there is a possibility of an undirected causal link between the degree of semantic tightness and the position of the verb in a sentence.

To summarize, the study shows that not all grammatical cues to subject and object are negatively correlated, as one would expect if one assumed that efficiency is directly reflected in relationships between aggregate typological variables. Still, there is a possibility that the trade-off between rigid word order and case marking is a manifestation of efficient behavior, and so is the weak correlation between tight semantics and the (final) position of the verb, where tight semantics helps to avoid costly reanalysis. The first claim is in fact supported by convergent evidence from artificial language learning experiments (Fedzechkina et al., 2016; Fedzechkina and Jaeger, 2020). Indeed, adult L2 learners avoid case marking in the presence of fixed word order. However, as was argued above, this manifestation of efficiency is only possible under certain conditions, which depend on the growing proportion of L2 users and possibly population size. Since the Subject and Object cues seem to be mostly influenced by the sociolinguistic factors, this leaves little space for potential manifestations of communicative efficiency.

A proper test of efficient behavior would require context-sensitive information about the joint distribution of linguistic cues, which also takes into account their diverse functions in discourse. This is difficult to do at the moment due to technical reasons, such as data sparseness and lack of reliable morphological annotation. Still, this article shows that a causal analysis of aggregate linguistic variables can be used to circumscribe the potential effects of communicative efficiency in language evolution. These results need further support from typological and experimental data, as well as from corpora representing other languages and registers.

## Data Availability Statement

The datasets presented in this study can be found in online repositories. The names of the repository/repositories and accession number(s) can be found below: https://github.com/levshina/SubjectObjectCues.

## Author Contributions

NL was responsible for the design of the study, data collection, statistical analysis, and interpretation.

## Conflict of Interest

The author declares that the research was conducted in the absence of any commercial or financial relationships that could be construed as a potential conflict of interest.

## Footnotes

1 Notably, Fenk-Oczlon and Fenk disagree on what makes word order more or less complex. This is symptomatic of complexity research, with many possible definitions.

2 I thank Mira Ariel (p.c.) for sharing this idea.

3 Thanks to Björn Wiemer (p.c.) for making me aware of this interesting feature.

4 Fenk-Oczlon and Pilz attribute the inverse correlation between word length and population words to a general increase in frequency of words when population increases, such that more frequent words will undergo formal reduction, according to Zipf’s law of abbreviation. But it is not clear how the higher frequency in the entire population would affect predictability of a word for individual speakers, who only communicate within their social networks. A more plausible explanation, in my view, is an increase in phonological inventories due to borrowings, which would allow for more monosyllabic words.

5 These results are for sentences with canonical (i.e., VO) order. When the order is non-canonical, the object index is always present.

6 This effect is restricted by different factors. In particular, it is observed when the free variation is between only two alternatives, and when adults reproduce already familiar input. When producing new utterances, adults fall back on their bias toward regularization (Wonnacott and Newport, 2005).

7 Note that languages with so-called free word order tend to have strong preferences with regard to the pragmatic role of the elements. For example, some polysynthetic languages tend to put newsworthy constituents first. This could also be an easy rule to acquire. This pattern is characteristic of languages with a full set of substantive bound pronouns referring to all core arguments attached to the verb in a rigid order, so the full noun phrases act like appositives to the pronominal affixes (Mithun, 1987). The question is then what serves as the main cues for Subject and Object – bound morphemes or nouns.

8 I thank Laura Becker (p.c.) for this idea.

## APPENDIX

**TABLE A1:** 95% confidence intervals around Spearman’s correlation coefficients based on 1,000 simulations.

	**Case marking**	**Tight semantics**	**Rigid word order**
Tight semantics	0.484, 0.499 *0.434, 0.448*		
Rigid word order	−0.670, −0.664 −*0.663*,−*0.657*	−0.162, −0.149 *0.257, 0.264*	
Verb between Subject and Object	−0.480, −0.469 −*0.229*,−*0.218*	−0.446, −0.440 −*0.278*,−*0.266*	0.269, 0.281 *0.011, 0.017*

*Upper numbers, non-partial correlations. Lower numbers, italics: partial correlations.*
